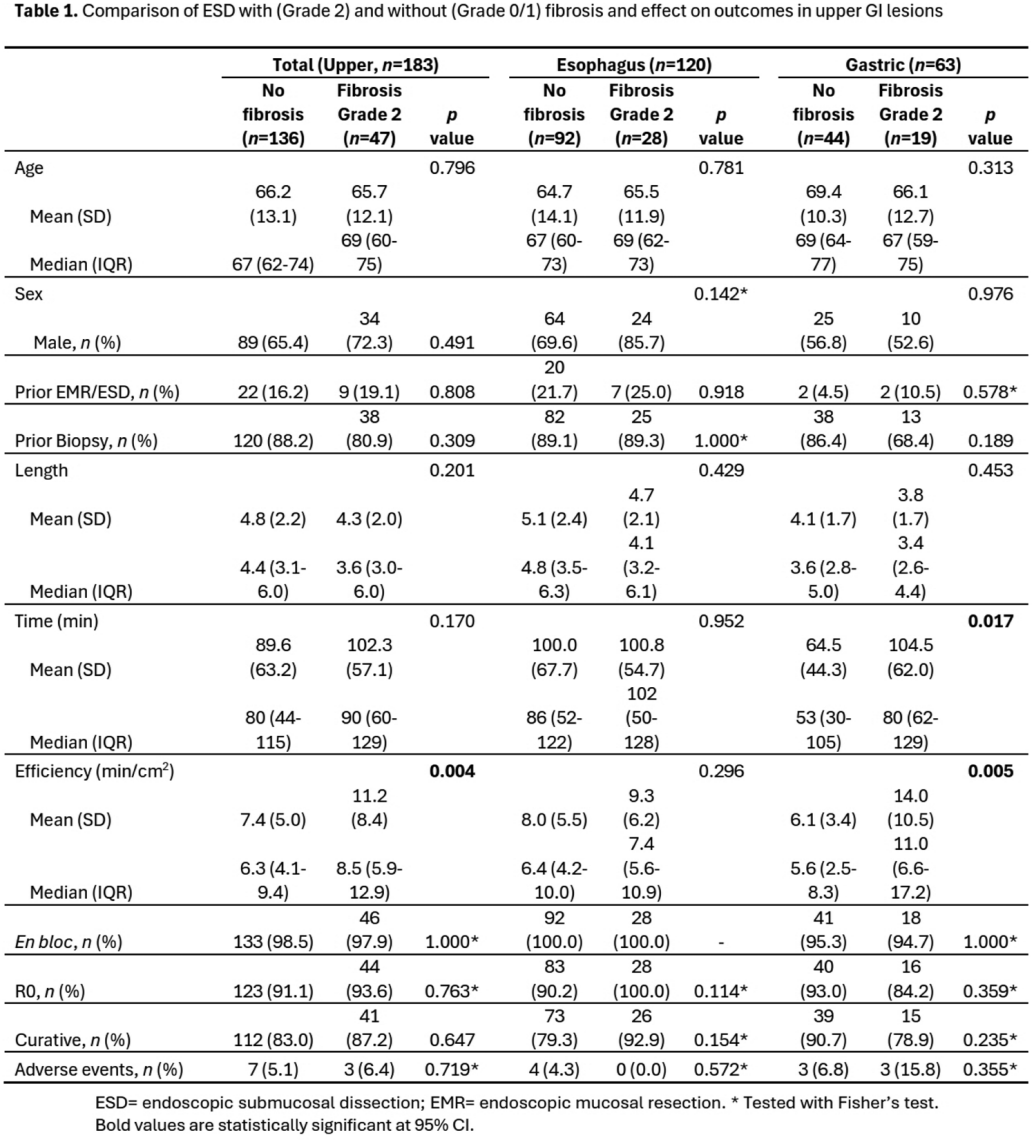# Poster Session I - A167 ROLE OF PREPROCEDURAL BIOPSIES IN UPPER GASTROINTESTINAL TUMORS UNDERGOING ENDOSCOPIC SUBMUCOSAL DISSECTION (ESD) AND IMPACT ON FIBROSIS AND RESECTION RATES

**DOI:** 10.1093/jcag/gwaf042.167

**Published:** 2026-02-13

**Authors:** C Ching Hui Yee, M Youssef, S Ghobrial, K Khalaf, R Bechara

**Affiliations:** McGill University, Montreal, QC, Canada; University of Toronto, Toronto, ON, Canada; University of Manitoba, Winnipeg, MB, Canada; Division of Gastroenterology, St. Michael’s Hospital, University of Toronto, Toronto, ON, Canada; Kingston Health Sciences Centre, Kingston, ON, Canada

## Abstract

**Background:**

Endoscopic submucosal dissection (ESD) is a minimally-invasive procedure widely used for resection of upper gastrointestinal lesions (GI) including esophageal and gastric tumors. Although preprocedural biopsies are routinely performed, their role is limited due to sampling error and risk of fibrosis which may impact technical outcomes of ESD.

**Aims:**

This study aims to evaluate the diagnostic performance of pre-ESD biopsies and the associated risk of fibrosis as well as the impact on technical outcomes of ESD.

**Methods:**

This is a retrospective cohort study of all patients who underwent ESD for upper GI lesions at Kingston Health Sciences Center (KHSC) between October 2016-September 2025. We analyzed clinical, endoscopic, and histopathologic characteristics of lesions as well as patient outcomes. Histological diagnoses were categorized using the WHO Classification criteria for GI lesions, 5th edition. Rates of concordance and upstaging were calculated comparing the initial and final resected pathology. Univariate analysis between the presence of fibrosis and technical outcomes such as resection time, procedural efficiency, en-bloc, R0, curative resections, and adverse events were used to calculate odds ratios with 95% confidence intervals. P-value of < 0.05 was considered statistically significant.

**Results:**

This cohort study included 183 patients with upper GI lesions (esophageal=120, gastric= 63) that underwent ESD at KHSC. 158/183 (86.3%) of patients had preprocedural biopsies prior to their ESD at KHSC. The overall histologic concordance rate between tissue biopsy and ESD specimens was 48.4% (38.8% for esophageal and 68.0% for gastric lesions). 46.4% (n = 71) of lesions were upstaged from initial biopsy with 84.5% (n = 60) of those final resections being neoplastic lesions. The risk of fibrosis was similar between patients with and without pre-ESD biopsies (23.9% vs 36.0%, p = 0.115). Lesions with fibrosis affected the procedural effiicency (mean 11.2 min/cm^2^ vs. 7.4 min/cm^2^, p = 0.026). However, the presence of fibrosis did not have a statistically significant impact on en-bloc resections (p = 1.00), R0 resections (p = 0.763), curative resections (p = 0.647), or on periprocedural adverse events (p = 0.719). Subgroup analysis showed that fibrosis did affect ESD efficiency in gastric lesions (14.0 min/cm^2^ vs 6.1 min/cm^2^, p = 0.005) but not in esophageal lesions (p = 0.296). Technical outcomes and adverse events remained consistent between site-specific analyses and the overall cohort.

**Conclusions:**

Preprocedural biopsies have poor concordance rate with post-ESD tissue histology. Biopsies of upper GI lesions do not seem to increase the risk of fibrosis. Fibrosis may limit ESD procedural efficiency but does not impact ESD technical outcomes or periprocedural adverse events.

**Funding Agencies:**

NoneUniversity of Toronto - Gastroenterology & Hepatology Department